# Cardioneuroablation in the Management of Vagally Mediated Bradyarrhythmias—A Comprehensive Review of Ongoing Randomized Controlled Trials

**DOI:** 10.3390/jcm14020592

**Published:** 2025-01-17

**Authors:** Przemysław Skoczyński, Sebastian Stec, Anna Ratajska, Magdalena Zając, Bruno Hrymniak, Anna Kustroń, Agnieszka Andrejków, Edyta Stodółkiewicz-Nowarska, Janusz Śledź, Dariusz Jagielski

**Affiliations:** 1 Department of Cardiology, Center for Heart Diseases, 4th Military Hospital, 50-981 Wrocław, Poland; 2 Department of Emergency Medicine, Wrocław Medical University, 50-368 Wrocław, Poland; 3 FESC Department of Cardiac Surgery and Transplantation, National Medical Institute of the Ministry of the Interior and Administration, 02-507 Warsaw, Poland; 4 ELMedica, EP-Network, 26-110 Skarzysko-Kamienna, Poland; 5 Psychological Therapeutic and Research Center, University Hospital No. 2, 85-168 Bydgoszcz, Poland; 6 Department of Humanization, Medicine and Sexology, Institute of Health Sciences, University of Zielona Góra, 65-516 Zielona Góra, Poland; 7 BioSphere Research and Trials, 02-507 Warsaw, Poland; 8 Clinical Research Support Center, 4th Military Hospital, 50-981 Wrocław, Poland; 9 CardioMedicum—Institute for Cardiovascular Research, 30-002 Cracow, Poland; 10 Faculty of Medicine, Wrocław University of Science and Technology, 50-370 Wroclaw, Poland

**Keywords:** cardioneuroablation (CNA), vagally mediated bradyarrhythmia

## Abstract

Cardioneuroablation is a rapidly developing procedure for the treatment of vagally mediated bradyarrhythmias. However, the lack of multicenter, randomized trials prevents it from being included in bradyarrhythmia treatment guidelines. So far, only one small, randomized study has been published assessing the effectiveness of this method in the treatment of reflex syncope. This is a brief review of ongoing randomized trials evaluating the effectiveness and safety of cardioneuroablation for the treatment of functional bradyarrhythmias.

## 1. Introduction

Cardioneuroablation (CNA) is a rapidly developing, percutaneous, catheter-based, minimally invasive electrophysiologic procedure for the treatment of vagally mediated bradyarrhythmias (VMBs) [1,2,3,4,5,6]. Several small, open-label cohort studies and a single small, randomized trial confirmed its overall 80–95% middle-term efficacy for a wide spectrum of VMBs, including sinus node dysfunction (SND), vagally mediated atrial fibrillation (AF), atrioventricular blocks (AVBs), cardioinhibitory (CI) or mixed (Mix) vasovagal/neurocardiogenic syncope (VVS/NCS), and CI or Mix carotid sinus syndrome or hypersensitivity (CSS/CSH) [1,2,3,4,5,6,7,8,9,10,11,12,13,14]. Moreover, CNA was documented to change the scenarios of clinical management in patients with class I, IIa, or IIb indications (according to ESC/EHRA/ACC/AHA/HRS guidelines) for pacemaker (PM) de novo implantation or PM replacement [5,6,7,8,9,10,11,12,13,14]. Although CNA is reported to be associated with high efficacy, low risk of complications, and significant improvement of patient-related outcomes (PROs), symptom management, and improvement in quality of life (QoL), especially in patients with VVS/NCS, this procedure is not yet recommended in current ESC/EHRA/ACC/AHA/HRS guidelines [1,2,3,4,5,6,7,8,9,10,11,12,13,14]. Herein, there is currently no clearly defined strategy for the clinical management of VMB. However, an alternative approach is supported when hypervagotonia is diagnosed as the main mechanism of bradycardia. A lack of randomized controlled trials (RCTs) is the main limitation to wide implementation of this technique in mainstream practice and guidelines [1,2,3,4,5,6,7,8,9,10,11,12,13,14]. Therefore, this experimental and innovative procedure is currently implemented “beyond guidelines” only in very advanced centers using a patient-oriented approach, shared decision making, and recruiting patients for ongoing clinical registries and RCTs. Several controversies exist in the field of CNA and the assessment of its efficacy and long-term safety. The indications for CNA in VMB are reported to be mainly recommended at the age of below 60 years; however, even in the elderly, CNA may be beneficial in properly selected patients [5]. A reduction in the gap in evidence-based medicine and clinical practice may only be achieved by large clinical registries with long-term follow-up and RCTs with or without sham procedures. Unfortunately, the Scientific Statement Cardioneuroablation for the Treatment of Reflex Syncope and Functional Bradyarrhythmias is based on several non-RCT trials and only two published RCTs to date [15]. Thus, it did not consider the potential impact of ongoing RCTs on future guidelines for syncope and VMBs. Updated future guidelines and broad implementation of the CNA procedure in all VMBs require further efforts and good-quality RCT. Therefore, this review will focus on summarizing ongoing trials investigating the use of CNA in various types of VMBs and VVS/NCS to evaluate whether these studies have the potential to influence future guidelines on syncope and VMBs.

## 2. Methods

We reviewed current registered and recruiting RCTs for CNA.

Before initiating the literature screening process, we established specific eligibility criteria, detailed as follows:

### 2.1. Participants

The included studies focused on participants diagnosed with VMBs, VVS, or vagal AF. Age, sex, and race were not considered exclusion criteria.

### 2.2. Intervention

The intervention under investigation was cardioneuroablation (CNA).

### 2.3. Study Design and Selection

**Figure 1:**
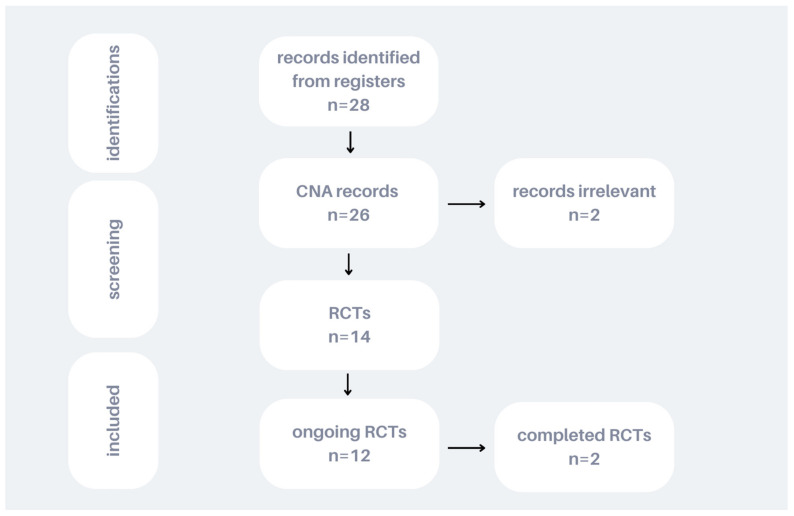
Flowchart illustrating the article selection process for the review.

Only ongoing randomized controlled trials (RCTs) were deemed eligible for inclusion. The study selection process is illustrated in Figure 1.

The non-RCTs regarding CNA were excluded from the review. These data were extracted from:

(1) ClinicalTrials.Gov (NCT) at http://www.clinicaltrials.gov (accessed on 1 June 2024);
(2) The European Union Clinical Trials Registered;
(3) The World Health Organization’s International Clinical Trials; and
(4) The International Standard Randomized Controlled Trial Number Register (ISRCTN) at http://www.controlled-trials.com (accessed on 1 June 2024).

The terms “cardioneuroablation” and “cardiac parasympathetic neuromodulation” were used to search for trials on these websites. The search period covered the time from the inception of these databases up to 1 June 2024.

The results of the search were then screened for clinical relevance and to determine if the trial was ongoing, recruiting patients, stopped, or had been completed. Special attention was paid to randomization, and the trial had at least one interventional arm. Once accepted for analyses, the trial data were evaluated for study design, methods, primary and secondary outcomes, and inclusion and exclusion criteria. Finally, a PubMed search was screened to validate whether there were protocols or primary results related to the ongoing studies.

### 2.4. Study Focus

The included studies evaluated the safety and efficacy of CNA in treating VVS, VMBs, and vagal AF.

## 3. Results

**Figure 2:**
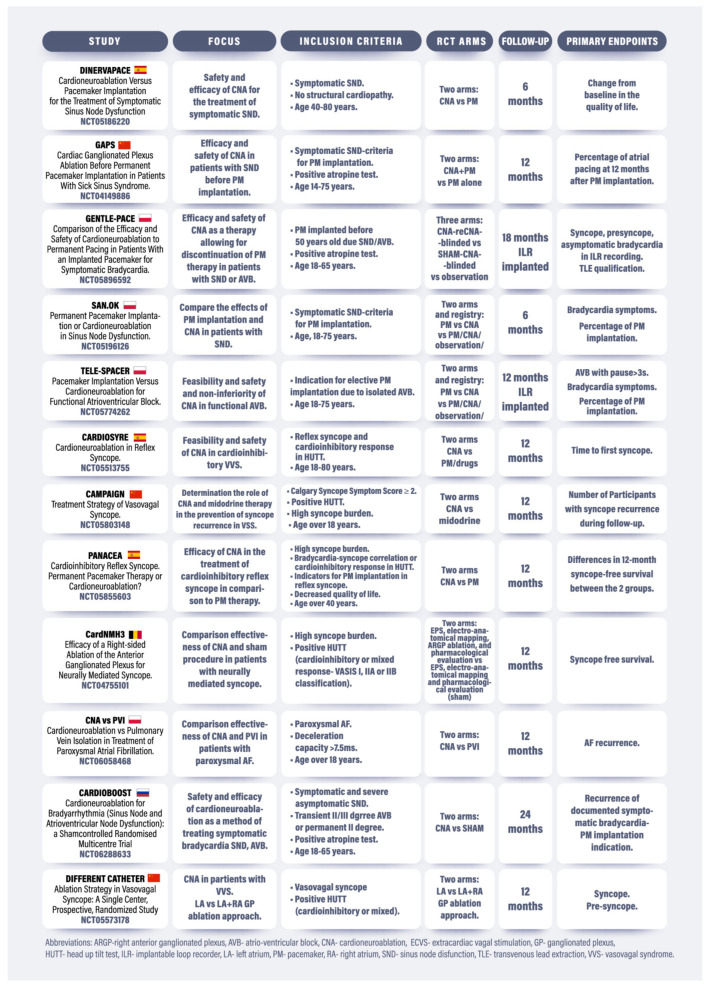
Summary of ongoing RCTs regarding CNA.

There were 12 RCTs registered with CNA, which had at least one interventional arm. The summary of the results is shown in Figure 2. No RCTs were international, and none involved a pediatric population. There were nine multicenter RCTs. There were three trials with the sham procedure (CardioNMH3, GENTLE-PACE, CARDIOBOOST). A single trial evaluates the management strategy for discontinuing permanent PM therapy. Three trials are planned to assess RCT arms and registry of patients who will not accept randomization. Almost half (5/12) of RCTs are focused on VVS/NCS. There are no single trials directly comparing CNA with PM in CSS/CSH, and only one small trial comparing CNA with catheter ablation in vagally mediated AF. The primary endpoints in most trials are a symptom-based approach and assessment of QoL. There are, however, only two trials with very long-term follow-ups (24 months). Only three RCTs use continuous ECG monitoring (ILR:2; PM:1) to validate the clinical endpoints of CNA [10,16,17,18,19,20,21,22,23,24,25,26]. No single trial has planned cost-effectiveness analysis as a secondary endpoint. Inclusion criteria of elderly patients (over age of 60 years) were reported in all 12 RCTs (Figure 2). The control group underwent various procedures depending on the type of study:

- VMBs and VVS: CNA vs. PM implantation or CNA vs. sham procedure; and
- Vagal AF: CNA vs. pulmonary vein isolation

The study protocols were independently published in the literature only in three studies (SAN.OK, GENTLE-PACE, TELE-SPACER) [10,18,20].

## 4. Discussion

Our review confirms that implementation of RCTs in the field of CNA is difficult and requires further progress and development. The analysis showed difficulties in the implementation of a true blinded approach, sham procedures, and comprehensive assessment prior to and after CNA (cardiovascular autonomic tests (CAT), telemedicine, ILR monitoring, and non-syncopal symptoms analyses) [1,2,3,4,5,6,7,8,9,10,11,12,13,14].

The only randomized studies published so far have assessed the effectiveness of CNA in CI/mix VVS/NCS. The ROMAN study proved the effectiveness of CNA in this group of patients, both in terms of preventing syncope and improving the quality of life (QoL) compared to the control group in which non-pharmacological conservative treatment was used. However, its main limitation is the small population of the study group (*n* = 24) and the control group (*n* = 24). Moreover, it was not blinded, the comparator was conservative treatment without pacemaker therapy and ILR monitoring, and the sham procedure was not used [4]. The ROMAN 2 study assessed the peri-procedural aspects of the extent of the CNA procedure, comparing the approach of only right atrial (RA) CNA vs. left atrial (LA) vs. biatrial CNA if the RA CNA or LA CNA approach alone was ineffective. The procedure’s effectiveness was assessed only peri-procedurally using extracardiac vagal nerve stimulation (ECVS). The greatest effectiveness was demonstrated in the group undergoing biatrial CNA. The effectiveness of CNA performed only in the right atrium was the lowest. The main limitation of this study is the short follow-up period [6]. One of the registered trials is a prospective study with ILR monitoring without randomization (Italian CNA, NCT05751330); therefore, it was excluded as non-RCT.

The more shared decision making is implemented in the field of invasive electrophysiology, the more patient-tailored management strategies are used for the individual patient, especially in the elderly population. As well as syncopal episodes in VVS/NCS/CSS, a wide range of symptomatic patients will be evaluated in ongoing trials, but non-syncopal groups require further progress.

Future trials should systematically validate the reasons for screening failure, randomization failure, complex substrate, and coincidence of intrinsic and extrinsic etiologies in a single patient, several PROs (including impact on occupational medicine, fitness to drive, sport medicine, and cognitive dysfunction), using CAT, and cost-effectiveness analysis. Thanks to Pachon’s invention and the implementation of CNA, ECVS, and other techniques, the focus on recognition of functional, vagally mediated bradyarrhythmia has already revolutionized the management of patients in the field of “bradycardiology” [1]. On the other hand, the experience from one of the first RCTs in symptomatic patients with SND (*n* =107, Theo-PACE with three equally distributed arms: elective PM implantation, theophylline oral treatment, and observation, which recruited fewer than 150 patients) has the most significant impact on the previous and current versions of ESC/EHRA/ACC/AHA/HRS guidelines [1,2,3,4,5,6,7,8,9,10,11,12,13,14,27].

CNA has been shown to improve syncope burden and VVS-related symptoms in single or multicenter clinical studies and one RCT. However, relatively small sample sizes and short follow-up periods (<3 years) might be insufficient to implement CNA and an autonomic approach in mainstream bradycardia management strategies. Moreover, a large group of patients with bradycardia and VMB not related to syncope has not yet been reported to spread the indications for bradycardia management to patients with borderline or weak indications for current PM therapy.

On the other hand, the chronic imbalance between sympathetic and parasympathetic drives after CNA raises concerns about the potential risks of CNA for long-term side effects of other cardiovascular disorders, including atrial and severe ventricular arrhythmias, inappropriate post-CNA sinus tachycardia, or other known or unpredicted complications of CNA.

The strength and quality of future recommendations will depend on CNA experts, clinical practitioners, and international authorities developing well-designed and good-quality multicenter registries and several multicenter and international RCTs. Appropriate education, patient association support, and social media campaigns should be made to encourage patients to enroll in these and future clinical trials. The evaluation of patients for CNA is complex; however, includes precise, patient-oriented, and preventive strategies to avoid symptoms, PM implantation, and other important factors for our patients’ PROs and influence of vagally mediated bradycardias and its treatment on QoLs [23,24,25].

The possibility of modifying the ESC/EHRA/ACC/AHA/HRS guidelines for treating vasovagal bradyarrhythmias will be fully justified when it becomes possible to compare the results of various clinical studies describing the use of CNA. To date, the completed and published studies contain different methodologies, inclusion and exclusion criteria, and endpoints—and, importantly, they did not include RCTs. The heterogeneity of the completed studies does not allow for a reliable meta-analysis but only a systematic review with a meta-analysis burdened with significant doubts, which does not provide a solid basis for formulating guidelines. RCTs are one of the essential conditions for conducting a reliable meta-analysis of data.

Four meta-analyses comparing the efficacy of CNA in VVS therapy have been registered in the PROSPERO register [28,29,30,31]. The endpoint of three of these meta-analyses will be time without syncope [29,30,31], prodromal symptoms [28,30], heart rate (HR), or heart rate variability (HRV) [29,31]. The analyses will examine proportions [29,30], RR [28], or weighted mean differences [31]. A meta-analysis [29] resulting from the work of an extended team [32] has been published, which, in the conclusion of comparing 14 studies and the treatment effects of 465 patients, emphasizes the necessity of conducting well-designed, double-blind, multicenter, sham-controlled, randomized clinical trials. Another meta-analysis formulates recommendations for CNA in VVS but stresses that further structured and comprehensive studies are needed [33]. The application of strict methodological criteria and multiple endpoints (not only regarding the effectiveness of the procedure but also the QoL of patients and the cost-effectiveness of the therapy) will allow for the development of guidelines from the perspective of nosology, pharmacoeconomics, and effectiveness for the psychosocial functioning of individuals with VVS. Due to the requirements of conducting a meta-analysis according to the PRISMA standard, the need to estimate (among other things) risk of bias, the design of the models used in the meta-analysis in comparing outcomes, a minimum of nine separate CTs—listed in Figure 2, registered intervention studies potentially meet the criteria for inclusion in the meta-analysis [34]. The published inclusion/exclusion criteria, length of follow-up, and primary endpoints suggest the use of a random effect model [35,36]. The registered studies cited in this article are sufficient in terms of the number of people undergoing the intervention (the CNA procedure will be performed in more than 750 people, and the total number of people participating in the studies exceeds 1400) to make sound conclusions about the efficacy of the method.

### 4.1. The Importance of Quality of Life in the Context of CNA

The role of patient perception of disease impact and response to therapy is increasingly recognized. Patient-reported outcomes, including health-related quality of life (HRQoL), provide information on the effectiveness of medical interventions that cannot be derived from objective clinical measurements [37]. Moreover, clinician-based health status measures, such as comorbidity, often miss the perspective of individuals and often correlate poorly with patient-reported outcomes such as HRQoL or subjective functioning [38]. There are few studies on the quality of life of patients treated with CNA. We know that a reduction in syncope is associated with increased patients’ quality of life [4], and multiple observational studies and one randomized trial have demonstrated the positive impact of CNA on quality of life [39]. The existing research results indicate that cardioneuroablation may be associated with intermediate-term improvement in QoL in patients with VVS. In this study, questionnaires on the subjective assessment of patient’s quality of life (the 36-Item Short Form Health Survey (SF-36) and EuroQol-5 dimension (EQ-5D-5L)) were used [40]. The SF-36 is a generic, coherent, and easily administered quality-of-life measure. The EQ-5D has two parts. The EQ-5D self-classifier asks patients to describe their health in terms of the level of problems (no/some/extreme) on each of five dimensions (mobility, self-care, usual activities, pain/discomfort, and anxiety/depression). The result is a health “profile”. The EQ-VAS is a vertical visual analog scale that takes values between 100 (best imaginable health) and 0 (worst imaginable health), on which patients provide a global assessment of their health [41]. Unfortunately, the methods have not been used to investigate the complex effects of CNA on the patient’s functioning, including his/her QoL. Addressing these difficulties, it seems essential to plan further studies in such a way as to explore these issues in more depth. Besides studying classic HR-QoL factors, it is important to identify patient-specific factors. A tremendous amount of work has been done by researchers analyzing the impact of syncope and presyncope on QoL and QoL domains, identifying key factors influencing QoL in patients with syncopal disorders, and combining available data to compare QoL between syncopal disorders and population normative data. The paper analyzes the impact of orthostatic syncope and presyncope on QoL, as measured by generic quality-of-life instruments [42]. The most effective approach appears to be combining condition-specific measures with generic ones. Both disease-specific and generic HR-QoL questionnaires are essential. Generic tools like the EQ-5D assess broader health dimensions, including physical and mental well-being [43]. There is a strong need for a questionnaire that could offer detailed insights into CNA’s impact on daily life by focusing on specific symptoms and patients’ needs and concerns [44]. Moreover, an intriguing question arises: Can patients’ quality of life be taken as a determinant of treatment effectiveness? Taking a patient-centered perspective, this seems obvious.

However, is it possible to treat patient quality of life as a secondary endpoint? Answering this question requires further research.

**Figure 3:**
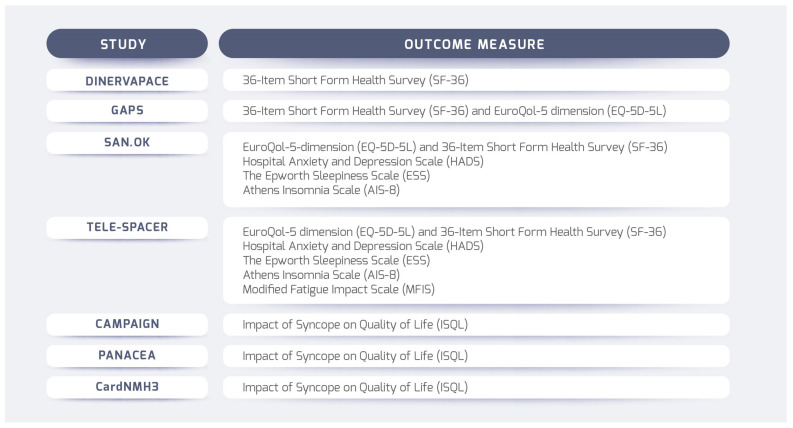
Methods used in RCTs currently registered in Clinical Trials in term of QoLs.

The methods used in the randomized trials currently registered in clinical trials are presented in Figure 3.

### 4.2. Current State of Cost Research on Cardioneuroablation

Currently, there are no specialized studies focusing exclusively on the costs of CNA. Instead, the literature primarily concentrates on the clinical aspects of this technique. However, the growing interest and development of this method suggest the need for future cost studies.

### 4.3. Cost Analysis of Other Medical Procedures

Economic studies on other medical procedures illustrate how these analyses can be valuable. For example, the study by Al-Khatib et al. (2018) [45] evaluates the guidelines for managing patients with arrhythmias. Despite the high initial costs of device implantation, long-term benefits may outweigh the costs, reducing long-term expenditures on hospitalizations and complication treatments. Additionally, the analysis highlights the long-term cost-effectiveness of CRT-D therapy compared to ICD, demonstrating that CRT-D therapy can be more cost-effective by reducing the need for hospitalizations and interventions related to heart failure [46]. Moreover, a report published in Poland in 2021 [47] examines the cost analysis of wireless pacemaker implantation. It emphasizes its potential economic benefits in reducing long-term treatment costs and lowering the risk of postoperative complications. In a similar vein, being an innovative method for treating arrhythmias, CNA could also offer significant economic benefits. Although no studies currently focus exclusively on its economic aspects, comparable treatment methods, such as those using pacemakers, suggest the possibility of cost savings through reduced complication rates and hospitalizations. Further research could explore whether similar savings are achievable with CNA, especially considering its potential effectiveness in reducing arrhythmias over the long term.

### 4.4. Missing Studies and Potential Valuable Contributions

There is a clear need for studies that thoroughly evaluate the economic aspects of CNA, particularly long-term cost comparisons of operations and treatments between CNA and traditional methods such as RF ablation or pacemaker implantation. Such a study could consider not only the direct costs of the procedures but also the indirect costs related to complications, patient quality of life, and long-term treatment efficacy.

In summary, despite the availability of economic studies on other treatment methods, more studies need to focus on the economic aspects of CNA. Future studies could provide valuable information to help achieve a more comprehensive assessment of this method in healthcare cost management.

## 5. Conclusions

The potential of the data inherent in the reports of RCTs on the efficacy of CNA, with the abundant representation of interventional studies and the large number of procedures performed, is a solid basis for thinking about revising the ESC/EHRA/ACC/AHA/HRS guidelines for the treatment of VMB. The well-documented studies and their meta-analysis should lead to a discussion and consideration of whether CNA represents a method to restore normative cardiovascular function in place of permanent treatment (PM/pharmacological/non-pharmacological), ultimately leading to a revision of the current guidelines.

The varying health outcomes of CNAs result in changes in patients’ functioning, including their quality of life. It is worth investigating this in-depth, using generic HR-QoL questionnaires and condition-specific measures together. Only then will we be able to comprehensively describe the effectiveness of CNA for treatment and patient functioning after the procedure.
